# Taxonomic relevance of petiole anatomical and micro-morphological characteristics of *Clematis* L. (Ranunculaceae) taxa from South Korea

**DOI:** 10.7717/peerj.11669

**Published:** 2021-06-29

**Authors:** Beom Kyun Park, Dong Chan Son, Balkrishna Ghimire

**Affiliations:** Division of Forest Biodiversity, Korea National Arboretum, Pocheon, South Korea

**Keywords:** Petiole morphology, *Clematis*, Trichomes, Vascular bundle, Infrageneric relationship, Taxonomy

## Abstract

We assessed the micro-morphological and anatomical structures of the petioles of 19 *Clematis* taxa from South Korea. The petiole surface features were observed with the help of stereomicroscopy and scanning electron microscopy, and the anatomical features are studied via microtomy and light microscopy. The results of this study showed that the presence/absence and abundance of trichomes, petiole cross-section outlines, upper surface wings and grooves, and the number of vascular bundles were useful for species discrimination in *Clematis*. Among the studied taxa, *C. hexapetala* was the only species with a glabrous petiole surface. Two types of trichomes were observed in the other 18 taxa: long, non-glandular and flagelliform trichomes and short, glandular capitate trichomes. We found four to six major vascular bundles and a maximum of eight interfascicular vascular bundles (*C. heracleifolia* and *C. urticifolia*) in the 19 taxa. A cluster analysis based on UPGMA identified six clusters with 18 nodes. Although the number of taxa investigated was limited, taxa from the sections *Tubulosae*, *Viorna*, and *Astragene* clustered with each other in the UPGMA phenogram due to the overall similarity of petiole features. Based on this observation, we can conclude that most of the petiole features are limited to the species level, and thus, the data obtained could be used as descriptive and/or diagnostic features for particular taxa, which may be useful for the investigation of problematic taxa in the genus.

## Introduction

Ranunculaceae is one of the larger families of eudicots, comprising nearly 2,500 species within 50–60 genera (Tamura, 1993; Hoot, Meyer & Manning, 2012; Wang et al., 2013). The family has been classified under the early branching order of eudicot Ranunculales (The Angiosperm Phylogeny Group, 2016). Ranunculaceae are distributed across the world and exhibit their greatest diversity in the temperate and cold regions of the Northern and Southern Hemispheres (Tamura, 1993). A number of classification models that consider morphological characters, molecular sequencing, and a combinations of both morphological and molecular data have been proposed for this family (Hutchinson, 1923; Janchen, 1949; Johansson & Jansen, 1993; Tamura, 1995; Hoot, 1995; Jensen et al., 1995; Ro, Keener & McPheron, 1997; Wang et al., 2009; Wang et al., 2013; Emadzade et al., 2010; Hoot, Meyer & Manning, 2012; Wang et al., 2014; Zhai et al., 2019). In addition to the various morphological criteria, the basic chromosome numbers and types of chromosomes have become reliable references that are compatible with the molecular phylogeny of Ranunculaceae (Gregory, 1941; Tamura, 1987; Ro, Keener & McPheron, 1997; Wang et al., 2009; Heywood et al., 2007).

Within Ranunculaceae, *Clematis* L. is classified under the tribe Anemoneae DC. in the subfamily Ranunculoideae Hutch. (Tamura, 1995). *Clematis* is one of the largest genera in the family, which comprises approximately 280–350 cosmopolitan species (Tamura, 1987; Tamura, 1995; Wang & Li, 2005). In Korea, Nakai (1952) reported 21 species and 14 varieties of *Clematis* in a synoptical sketch of Korean flora, but Lee (1967) later identified 16 species, 11 varieties, and five forma in the genus. In the book New Flora of Korea, Lee (2007) described 18 taxa, including *C. taeguensis* Y. Lee, which was first described by Lee (1982). The Korea National Arboretum (2017) recently listed 17 species and five varieties of *Clematis* in the Checklist of Vascular Plants in Korea, whereas Kim (2017) described 13 species and seven varieties within the genus in The Flora of Korea. After a careful review of Lee (2007); Chang, Kim & Chang (2011), the Korea National Arboretum (2017), and Kim (2017),we included 16 species and three varieties in this study.

Due to the vast morphological disparity among its species *Clematis* has been a subject of investigation since the early 19th century and has been subjected to several infrageneric revisions. Several systematic studies based on the anatomy of different parts, palynology, and cytology of *Clematis* have been carried out (Tobe, 1974; Tobe, 1980a; Tobe, 1980b; Tobe, 1980c; Tobe, 1980d; Essig, 1991; Zhang & He, 1991; Yano, 1993; Yang & Moore, 1999; Shi & Li, 2003; Xie & Li, 2012; Ghimire et al., 2020). The morphological characters that have been extensively studied and considered in the infrageneric classifications of *Clematis* include the habit, seed germination, seedling phyllotaxy, leaf structure, inflorescence vertical structure, floral morphology, and pollen and achene morphology (see Wang & Li, 2005).

The importance of the nodal and petiolar anatomy in intergeneric and familial taxonomy has been extensively studied (Howard, 1962; Howard, 1979; Schofield, 1968; Dickison, 1969; Dickison, 1980; Datta & Dasgupta, 1979). The middle portion of the petiole is considered the most stable zone, from which even a single section can be taken for comparative purposes (Metcalfe & Chalk, 1979). In addition, the complex vascular systems of the petiole provide a range of diagnostic structures that can be useful for taxonomic treatment at any rank (Solereder, 1908; Metcalfe & Chalk, 1979; Ashton, 1982; Dehgan, 1982; Rojo, 1987; Pedri, Hussin & Latiff, 1991; Kamel & Loufty, 2001; Kocsis & Borhidi, 2003; Talip et al., 2016; Talip et al., 2017). Within Ranunculaceae, few taxonomic-related investigations based on the anatomy of petioles, nodes, and stems have been carried out (Worsdell, 1908; Tamura, 1962; Oh, 1971; Kavathekár & Pillai, 1976; Tobe, 1979; Tobe, 1980a; Tobe, 1980b; Tobe, 1980c; Tobe, 1980d; Kökdil et al., 2006; Gostin, 2011; Novikoff & Mitka, 2015). Unfortunately, studies pertaining to the petiole morphology and anatomy of *Clematis*, as one of the most morphologically diverse and taxonomically complicated genera within Ranunculaceae, are very rare in the literature. In an anatomical study of *Clematis* in Korea, only Oh (1971) provided remarks on the petiole anatomy of nine Korean species.

In this study, we provide a comprehensive investigation of the petiole micromorphology and anatomy of 19 *Clematis* taxa distributed in Korea. The primary objective of this study was to investigate the petiole micromorphological and anatomical structure of the included taxa in detail and to evaluate the implications of petiolar characters for species delimitation. We attempted to compare our results with those obtained for previously studied species and summarized them to reach taxonomic conclusions.

## Materials and Methods

### Plant materials

The names of the investigated species and their voucher numbers are provided in Table 1. Formal identification of the plant taxa was carried out by a group of plant taxonomists, including Dr. Dong Chan Son (one of the authors) in the Korea National Arboretum. The voucher specimens were deposited in the herbarium of the Korea National Arboretum (KH). Data were collected as previously described in Ghimire et al. (2020)

**Table 1:** Name of taxa with voucher number and collection information with different classifications.

Lehtonen, Christenhusz & Falck (2016)	**Tamura (1987)**	**Johnson (1997)**	Wang & Li (2005)	**Taxon**	**Locality**	**Voucher No.**
Clade C	Sect. *Clematis*	Sect. *Clematis*	Sect. *Clematis*	*C. apiifolia* DC.	Mt. Sinbul, Icheon-ri, Sangbuk-myeon, Ulju-gun, Ulsan, Korea	Sinbulsan-190911-001
Clade C	Sect. *Clematis*	Sect. *Clematis*	Sect. *Clematis*	*C. brevicaudata* DC.	Unchi-ri, Sindong-eup, Jeongseon-gun, Gangwon-do, Korea	Unchiri-191007-001
Clade C	Sect. *Clematis*	Sect. *Clematis*	Sect. *Clematis*	*C. trichotoma* Nakai	Mt. Sinbul, Icheon-ri, Sangbuk-myeon, Ulju-gun, Ulsan, Korea	Sinbulsan-190911-001
Clade K	Sect. *Flammula*	Sect. *Flammula*	Sect. *Clematis*	*C. taeguensis* Y. Lee	Gyuram-ri, Jeongseon-eup, Jeongseon-gun, Gangwon-do, Korea	Gyuramri-190818-001
Clade K	Sect. *Angustifolia*	Sect. *Flammula*	Sect. *Clematis*	*C. hexapetala* Pall.	Ho-ri, Palbong-myeon, Seosan-si, Chungcheongnam-do, Korea	Hori-190809-001
Clade K	Sect. *Flammula*	Sect. *Flammula*	Sect. *Clematis*	*C. terniflora* DC.	Jukpo-ri, Dolsan-eup, Yeosu-si, Jeollanam-do, Korea	Dolsando-191004-002
Clade K	Sect. *Flammula*	Sect. *Flammula*	Sect. *Clematis*	*C. terniflora* var. *mandshurica* (Rupr.) Ohwi	Namhansanseong Fortress, Sanseong-ri, Namhansanseong-myeon, Gwangju-si, Gyeonggi-do, Korea	Namhansanseong-190809-001
Clade C	Sect. *Tubulosae*	Sect. *Tubulosae*	Sect. *Tubulosae*	*C. heracleifolia* DC.	Sihwa Lake, Munho-ri, Namyang-eup, Hwaseong-si, Gyeonggi-do, Korea	Sihwaho-190921-016
Clade C	Sect. *Tubulosae*	Sect. *Tubulosae*	Sect. *Tubulosae*	*C. urticifolia* Nakai ex Kitag.	Mt. Gariwang, Sugam-ri, Bukpyeong-myeon, Jeongseon-gun, Gangwon-do, Korea	Gariwangsan-191007-001
Clade C	Sect. *Tubulosae*	Sect. *Tubulosae*	Sect. *Tubulosae*	*C. takedana* Makino	Sihwa Lake, Munho-ri, Namyang-eup, Hwaseong-si, Gyeonggi-do, Korea	Sihwaho-190921-001
Clade L	Sect. *Viticella*	Sect. *Viticella*	Sect. *Viticella*	*C. patens* C.Morren & Dence.	Mt. Johang, Samsong-ri, Cheongcheon-myeon, Goesan-gun, Chungcheongbuk-do, Korea	Johangsan-170831-049
Clade K	Sect. *Pterocarpa*	Sect. *Pterocarpa*	Sect. *Pterocarpa*	*C. brachyura* Maxim.	Seondol, Bangjeol-ri, Yeongwol-eup, Yeongwol-gun, Gangwon-do, Korea	Seondol-190719-001
Clade I	Sect. *Meclatis*	Sect. *Meclatis*	Sect. *Meclatis*	*C. serratifolia* Rehder	Gasong-ri, Dosan-myeon, Andong-si, Gyeongsangbuk-do, Korea	Gasongri-191007-001
Clade L	*Sect. Viorna*	*Sect. Viorna*	*Sect. Viorna*	*C. fusca* Turcz.	Mt. Cheongtae, Sapgyo-ri, Dunnae-myeon, Hoengseong-gun, Gangwon-do, Korea	Cheongtaesan-190819-001
Clade L	*Sect. Viorna*	*Sect. Viorna*	*Sect. Viorna*	*C. fusca* var. *flabellata* (Nakai) J. S. Kim	Eundae-bong, Gohan-ri, Gohan-eup, Jeongseon-gun, Gangwon-do, Korea	Eundaebong-190818-001
Clade L	*Sect. Viorna*	*Sect. Viorna*	*Sect. Viorna*	*C. fusca* var. *violacea* Maxim.	Mt. Baekhwa, Mawon-ri, Mungyeong-eup, Mungyeong-si, Gyeongsangbuk-do, Korea	Mungyeongsi(Mawonri, Baekhwasan-150707-007
Clade H	Sect. *Atragene*	Sect. *Atragene*	Sect. *Atragene*	*C. calcicola* J. S. Kim	Mt. Deokhang, Daei-ri, Singi-myeon, Samcheok-si, Gangwon-do, Korea	Deokhangsan-190818-001
Clade H	Sect. *Atragene*	Sect. *Atragene*	Sect. *Atragene*	*C. koreana* Kom.	Mt. Hambaek, Gohan-ri, Gohan-eup, Jeongseon-gun, Gangwon-do, Korea	Hambaeksan-190818-001
Clade H	Sect. *Atragene*	Sect. *Atragene*	Sect. *Atragene*	*C. ochotensis* (Pall.) Poiret	Mt. Gariwang, Sugam-ri, Bukpyeong-myeon, Jeongseon-gun, Gangwon-do, Korea	Gariwangsan-190819-007

### Stereo and scanning electron microscopy

The petiole morphology, including the indumentum, trichome type and abundance, and upper surface groove was observed under a stereomicroscope and a scanning electron microscope (SEM). A Leica MZ16 FA microscope (Leica Microsystems GmbH, Wetzlar, Germany) was used for the observations and digital images of the best-represented part of the petiole were taken with a Leica DFC420 C multifocal camera attached to the microscope. Before SEM imaging, petiole pieces were immersed in 100% ethanol and sputter-coated with gold in a KIC-IA COXEM Ion-Coater (COXEM. Co., Ltd., Daejeon, Korea). SEM imaging was carried out with a COXEM EM-30 PLUS+ table scanning electron microscope (COXEM) at 20 kV at the seed testing laboratory of the Korea National Arboretum.

### Microtome and light microscopy

At least three petioles of each taxon were subjected to microtome sectioning according to the following procedure used in Ghimire et al. (2020). Freshly collected leaf petioles were fixed in formalin, acetic acid, and 50% ethyl alcohol (FAA) at a ratio of 5:5:90 for one week and preserved in 50% ethyl alcohol. During the experiment, the preserved petioles were cut into small pieces (approximately two mm) and dehydrated with an ethanol series (50, 70, 80, 90, 95 and 100%). After complete dehydration, the petiole pieces were infiltrated with ethanol/Technovit mixtures (3:1, 1:1, 1:3, and 100% Technovit) and then embedded in Technovit 7100 resin. The embedded materials were cut into serial sections of 4–6 µm thickness using a Leica RM2255 rotary microtome (Leica Microsystems GmbH, Wetzlar, Germany) with disposable blades and attached to a glass slide. The slides were dried using an electric slide warmer for 12 h. The dried slides were stained with 0.1% toluidine blue ‘O’ for 60–90 s, rinsed with water and dried again with the slide warmer for at least 6 h to remove any remaining water (Johansen, 1940). The stained slides were then mounted with Entellan (Merck Co., Darmstadt, Germany) and later examined under a Leica DM3000 LED (Leica Microsystem, Wetzlar, Germany). Photomicrographs were taken with a scientific CMOS camera. Multiple image alignment was performed using Photoshop CS for Windows 2010.

### Morphometry and data analysis

Thirteen quantitative characters were categorized and coded binary and/or multistate. The character states and their codes are provided in Supplementary File S1. Principal component analysis (PCA) and cluster analysis using the unweighted pair group (UPGMA) clustering method using the Gower general similarity coefficient were carried out with MultiVariate Statistical Package 3.1 software (MVSP Version 3.1) (Kovach, 1999).

## Results

**Figure 1:**
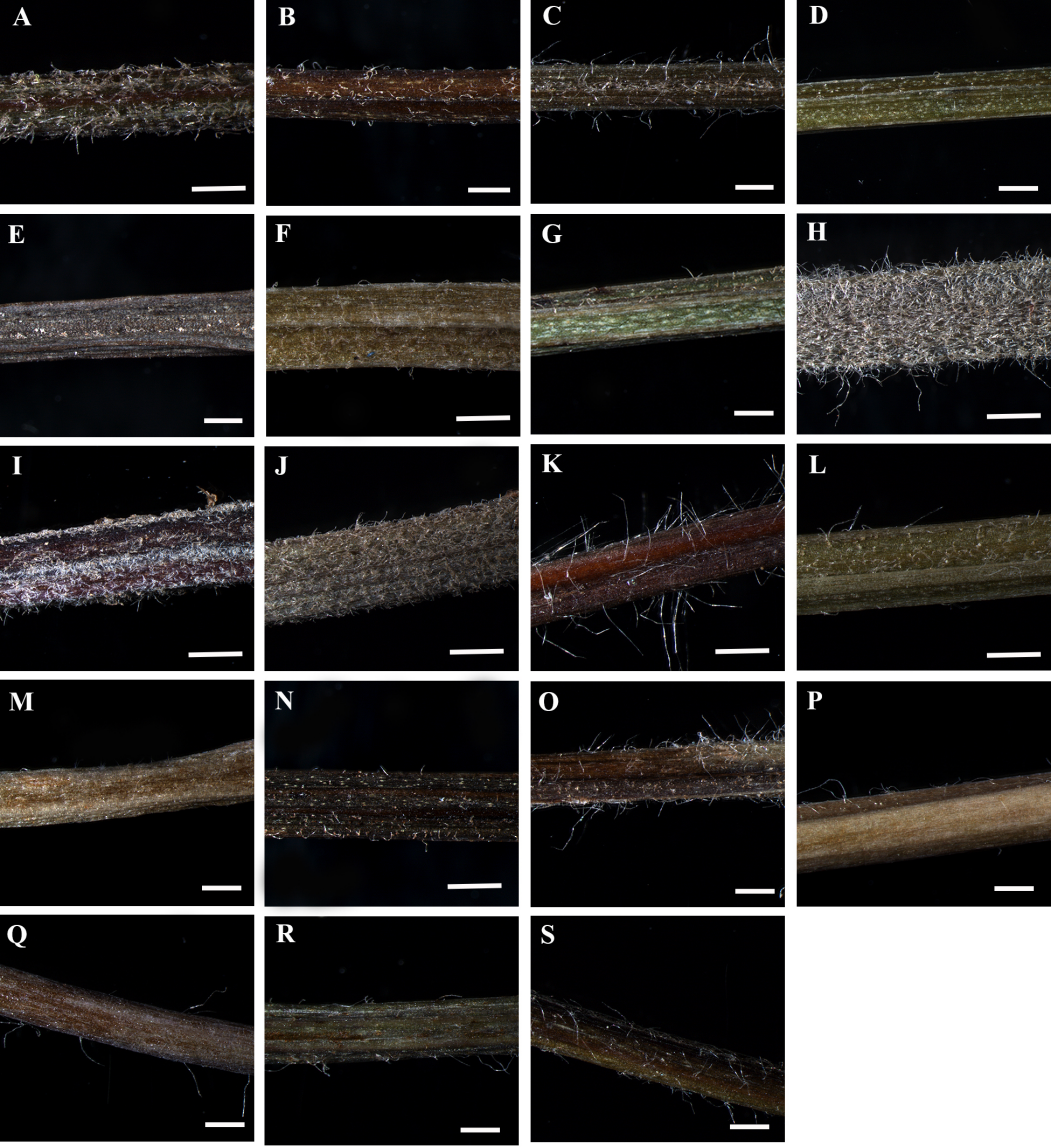
Petiole of *Clematis* under stereomicroscope. (A) *C. apiifolia*. (B) *C. brevicaudata*. (C) *C. trichotoma*. (D) *C. taeguensis.* (E) *C. hexapetala*. (F) *C. terniflora*. (G) *C. terniflora* var.* mandshurica*. (H) *C. heraclefolia*. (I) *C. urticifolia*. (J) *C. takedana*. (K) *C. patens*. (L) *C. brachyura*. (M. *C. serratifolia*. (N) *C. fusca* var. *fusca*. (O) *C. fusca* var. *flabellata*. (P) *C . fusca* var. *violacea*. (Q) *C. calcicola*. (R) *C. koreana*. (S) *C. ochotensis*. Scale bars: one mm.

**Figure 2:**
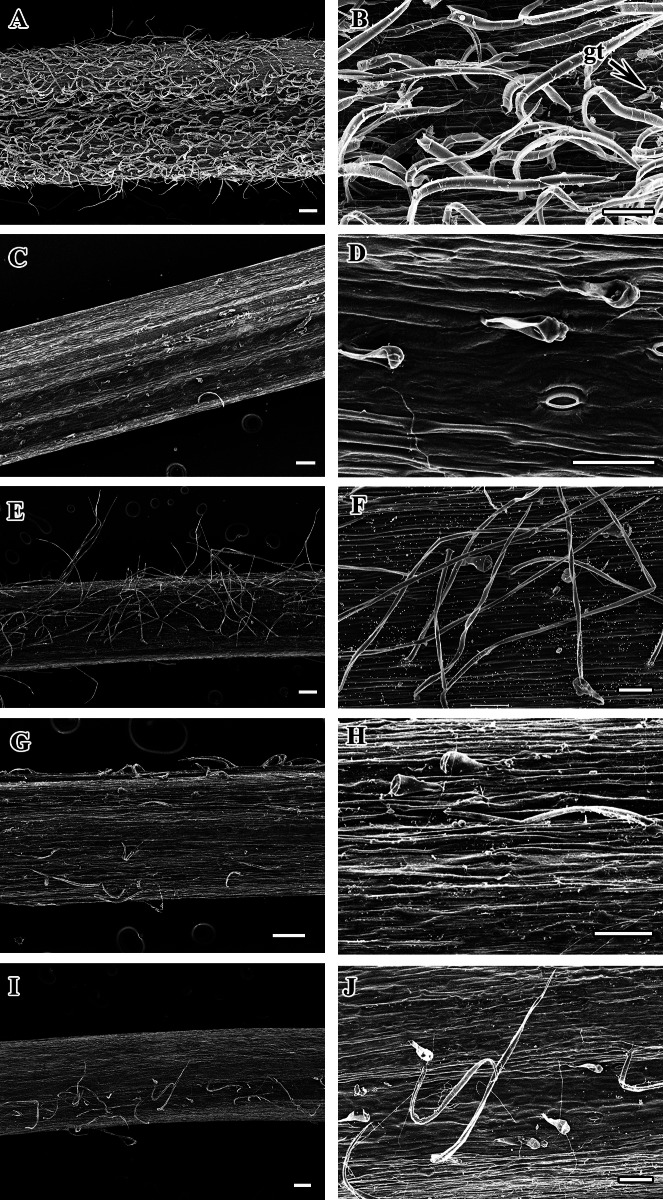
Scanning electron micrograph of petiole of *Clematis*. (A–B). *C. heraclefolia*. (C–D). *C. taeguensis.* (E–F). *C. patens*. (G–H). *C. brevicaudata*. (I–J). *C . fusca* var.* violacea*. Abbreviations: gt, glandular trichome. Scale bar: 200 µm (A, C, E, G, I), 100 µm (B, D, F, H, J).

**Figure 3:**
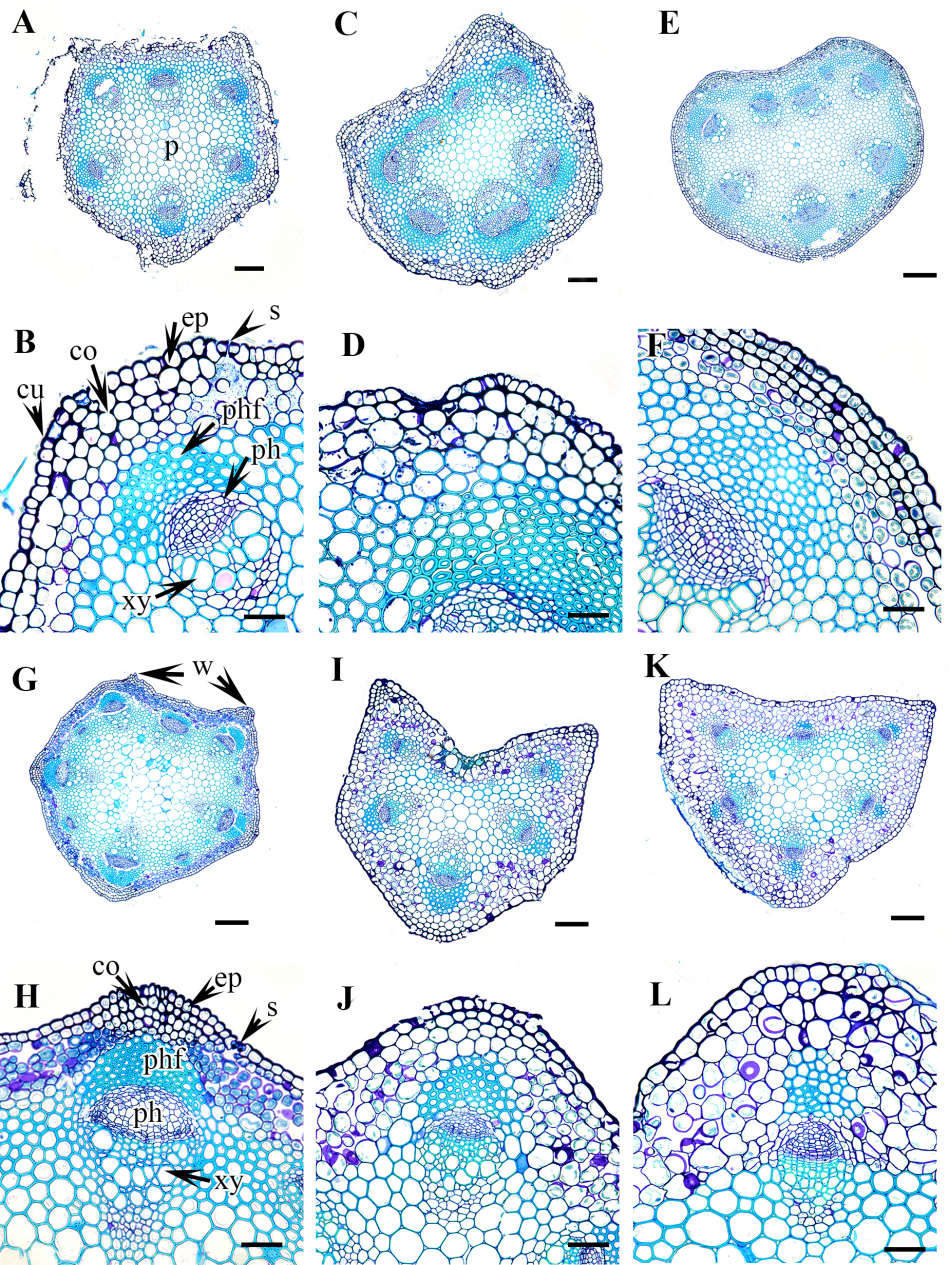
Cross section of petiole of *Clematis.*. (A–B) *C. apiifolia*. (C–D) *C. brevicaudata*. (E–F) *C. trichotoma*. (G–H) *C. taeguensis* (I–J) *C. hexapetala*. (K–L) *C. terniflora*. Abbreviations: co, collenchyma; cu, cuticle; ep, epidermis; ph, phloem; phf, phloem fiber; s, stomata; xy, xylem. Scale bars: 50 µm (B, D, J), 75 µm (F, H, L), 100 µm (A, C, I), 200 µm (E, G, K).

**Figure 4:**
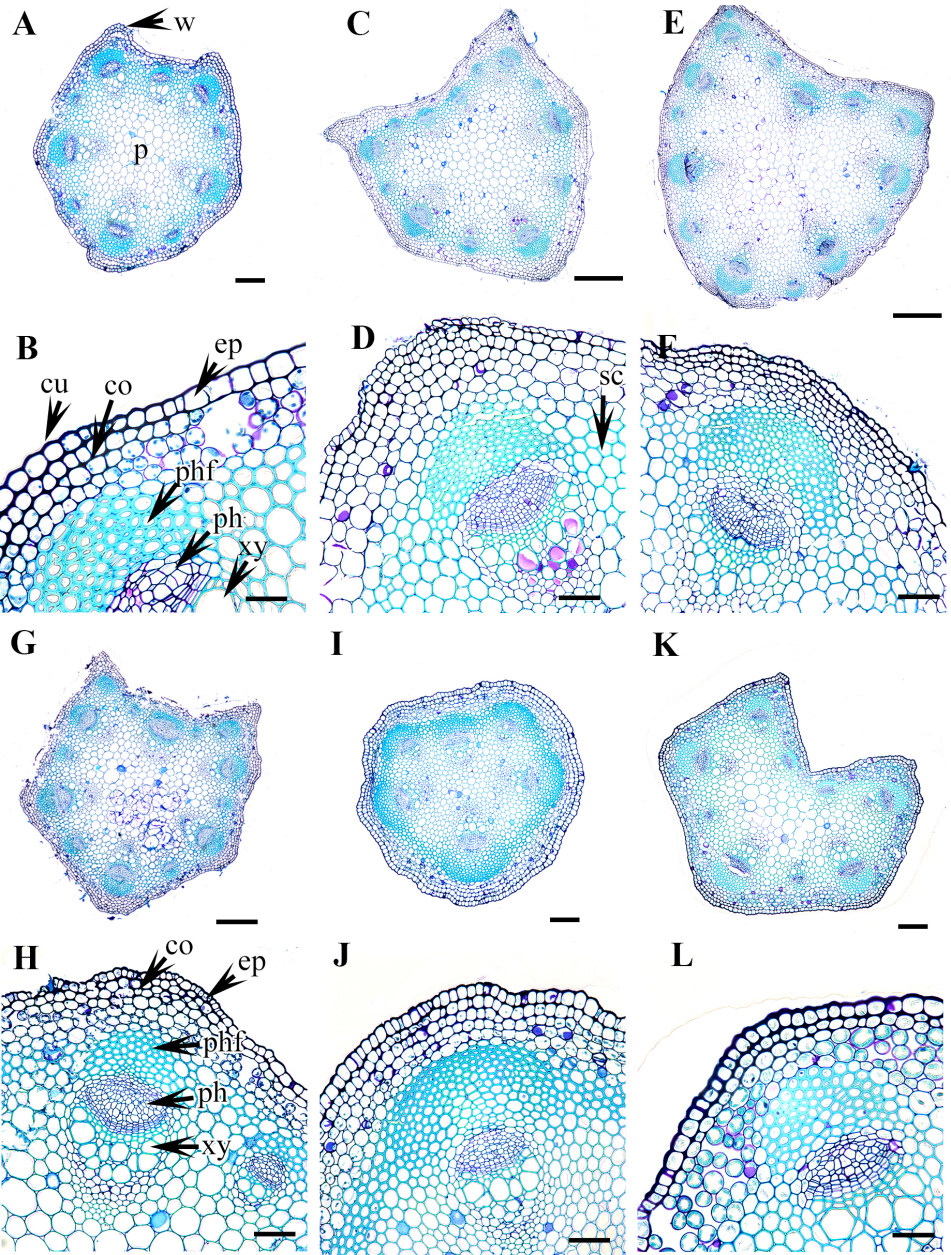
Cross section of petiole of *Clematis.*. (A–B). *C. terniflora* var.* mandshurica*. (C–D) *C. urticifolia*. (E–F) *C. heraclefolia*. (G–H) *C. takedana*. (I–J) *C. patens*. (K–L) *C. brachyura*. Abbreviations: co, collenchyma; cu, cuticle; ep, epidermis; ph, phloem; phf, phloem fiber; s, stomata; sc, sclerenchyma; xy, xylem. Scale bars: Scale bars: 50 µm (B, J, L), 100 µm (A, I, K, F, D, H), 500 µm (C, E, G).

**Figure 5:**
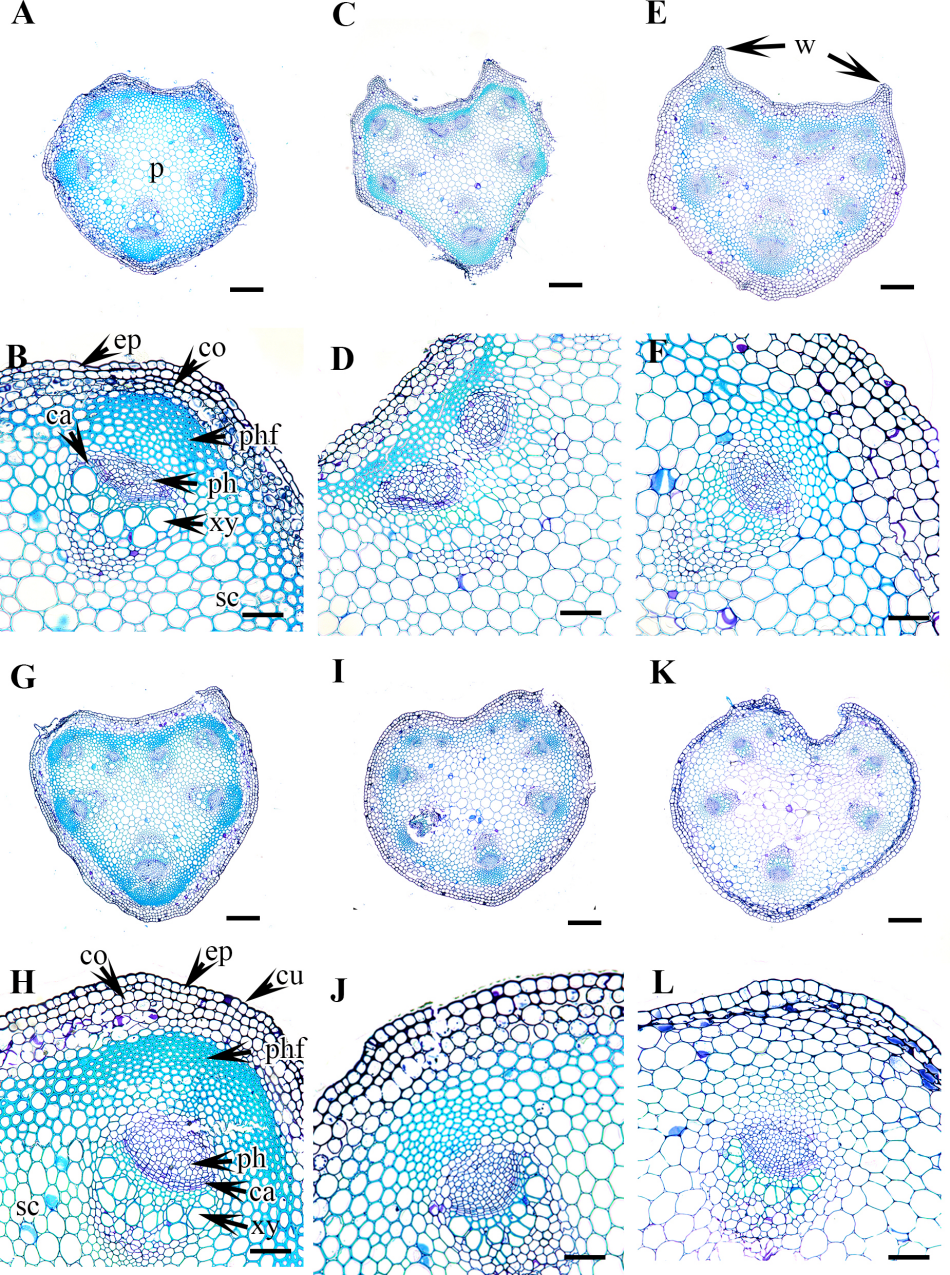
Cross section of petiole of *Clematis.*. (A–B). *C. serratifolia*. (C–D) *C. fusca* var. *fusca*. (E–F) *C. fusca* var. *flabellata*. (G–H) *C . fusca* var. *violacea*. (I–J) *C. calcicola*. (K–L) *C. koreana*. Abbreviations: co, collenchyma; cu, cuticle; ep, epidermis; ph, phloem; phf, phloem fiber; s, stomata; xy, xylem. Scale bars: 75 µm (B, D, F, H, J, L), 200 µm (A, C, E, G, I, K).

**Figure 6:**
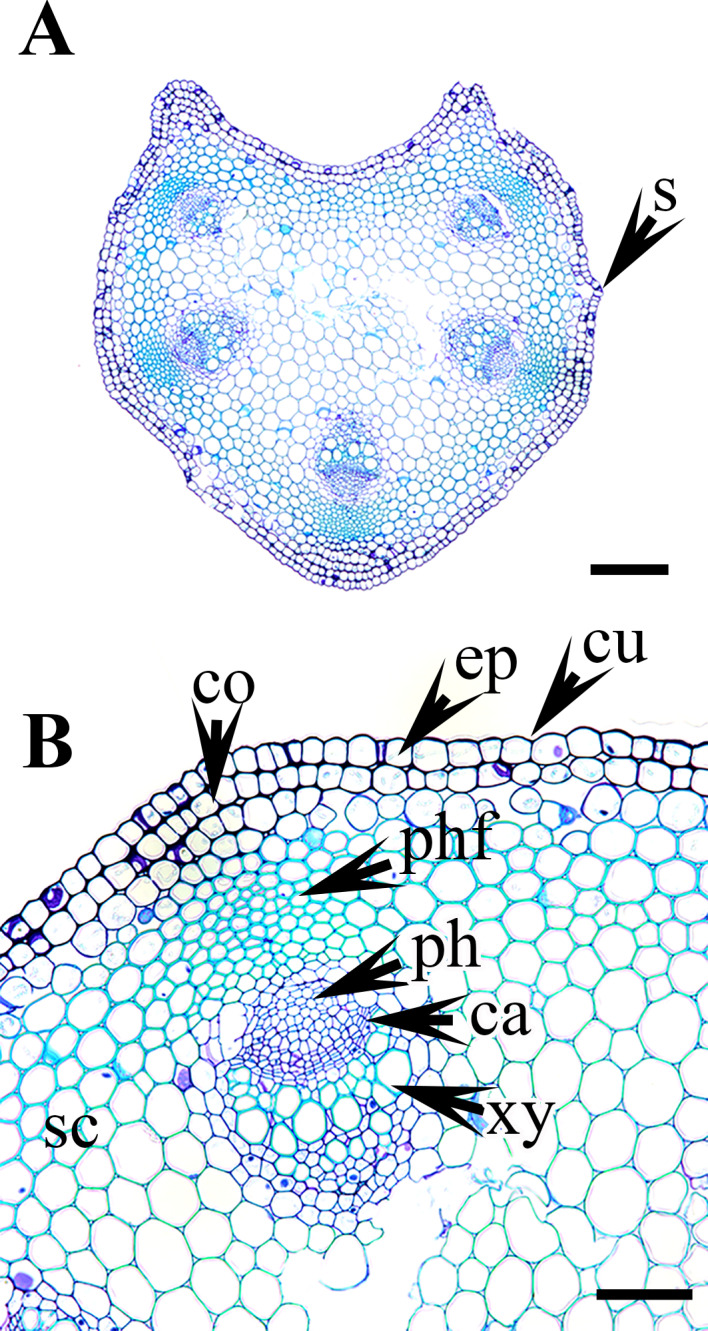
Cross section of petiole of *Clematis.*. (A–B). *C. ochotensis*. Abbreviations: co, collenchyma; cu, cuticle; ep, epidermis; ph, phloem; phf, phloem fiber; s, stomata; xy, xylem. Scale bars: 75 µm (B), 200 µm (A).

The morphological and anatomical characters of *Clematis* petioles observed in this study include the petiole indumentum, trichome type and abundance, petiole outline in cross-section (CS), upper surface wings and groove of the petiole, sclerenchyma region, and vascular bundles. All the characters are summarized in Table 2. Selected images of the petioles are provided in Figs. 1–6. The morphological and anatomical features of the petiole are comprehensively described below.

### Petiole surface and trichomes

The petiole surface of the studied species is pubescent except in *Clematis hexapetala*, which has an almost glabrous surface (a few trichomes occur in the region from which leaflets arise) (Table 2, Figs. 1A–1S). *Clematis taeguensis* and *C. serratifolia* have subglabrous petiole surfaces, and only a few trichomes occur on the petioles of these species. Two types of trichomes are observed on the petiole: long, non-glandular, flagelliform trichomes and short, glandular, capitate trichomes (Figs. 1A–1S, 2A–2J). Glandular trichomes are usually distributed in the upper surface groove. In some species such as *C. terniflora*, *C. terniflora* var. *mandshurica*, *C. brachyura*, *C. fusca* var. *fusca*, and *C. fusca* var. *violacea*, non-glandular trichomes are concentrated only on the upper surface groove. The pubescent species can be categorized as ‘villous’, which are covered with long, soft, and dense hairs (i.e., *C. apiifolia*, *C. brevicaudata*, *C. heracleifolia*, *C. urticifolia*, and *C. takedana*), or ‘pilose’, which are covered with soft, weak, thin, and separated hairs, as found in the rest of the species (i.e., *C. trichotoma*, *C. terniflora*, *C. terniflora* var. *mandshurica*, *C. patens*, *C. brachyuran*, *C. fusca* var. *fusca*, *C. fusca* var. *flabellata,C. fusca* var. *violacea*, *C. koreana*, and *C. ochotensis*). The non-glandular trichomes are either unicate (*C. apiifolia*, *C. brevicaudata*, *C. heracleifolia*, *C. urticifolia*, and *C. takedana*) or flabelliform (all other species except *C. hexapetala*). Based on the trichome density per unit area, the trichome abundance was categorized as high, medium, or low.

**Table 2:** Morphological and anatomical features of petiole of *Clematis* species.

Taxon	Petiole surface	Non-glandular trichomes	Glandular trichomes	Trichome abundance	Petiole outline in cross section	Upper surface wings
*C. apiifolia*	Villous	Unicate	Present	High	Pentagonal	Inconspicuous/ conspicuous
*C. brevicaudata*	Villous	Unicate	Present	Medium	U-shaped	Inconspicuous
*C. trichotoma*	Pilose	Flagelliform	Present	Medium	U-shaped	Inconspicuous
*C. taeguensis*	Subglabrous/pilose	Flagelliform	Present	Low	Pentagonal	Conspicuous
*C. hexapetala*	Glabrous	Absent	Absent	None	U-shaped/ pentagonal	Conspicuous
*C. terniflora*	Pilose	Flagelliform	Present	Low	Semi-circular/ U-shaped	Inconspicuous/ conspicuous
*C. terniflora* var. *mandshurica*	Pilose	Flagelliform	Present	Low	Pentagonal	Conspicuous
*C. urticifolia*	Villous	Unicate	Present	High	Pentagonal	Conspicuous
*C. heracleifolia*	Villous	Unicate	Present	High	U-shaped	Inconspicuous
*C. takedana*	Villous	Unicate	Present	High	Pentagonal	Conspicuous
*C. patens*	Pilose	Flagelliform	Present	Medium	U-shaped	Inconspicuous
*C. brachyura*	Pilose	Flagelliform	Present	Medium (in upper surface groove)	Pentagonal	Conspicuous
*C. serratifolia*	Subglabrous/pilose	Flagelliform	Absent	Low	U-shaped/ pentagonal	Conspicuous
*C. fusca* var. *fusca*	Pilose	Flagelliform	Present	Low	Pentagonal	Conspicuous
*C. fusca* var. *flabellata*	Pilose	Flagelliform	Present	Medium	U-shaped	Conspicuous
*C. fusca* var. *violacea*	Pilose	Flagelliform (in groove)	Present	Low	U-shaped	Conspicuous
*C. calcicola*	Subglabrous/pilose	Flagelliform	Absent	Low	U-shaped	Inconspicuous/ conspicuous
*C. koreana*	Pilose	Flagelliform	Present	Medium	U-shaped	Conspicuous
*C. ochotensis*	Pilose	Flagelliform	Present	Medium	U-shaped	Conspicuous

Taxon	Upper surface groove	Phloem fiber cap	Interfascicular sclerenchyma	MVB	IVB	TVB	BVG
*C. apiifolia*	Flattened/Sub-flattened	Medium, 5-10 layers	<5 layers	6	0	6	1
*C. brevicaudata*	Sub flattened	Large, >10 layers	>10 layers	5	2 to 3	7 to 8	2
*C. trichotoma*	Sub flattened	Large, >10 layers	5-10 layers	5	2	7	2
*C. taeguensis*	Sub flattened	Large, >10 layers	>10 layers	6	4	10	1
*C. hexapetala*	V-shaped	Medium, 5-10 layers	<5 layers	5	0	5	0
*C. terniflora*	Flattened/Sub flattened	Small, <5 layers	<5 layers	6	0	6	1
*C. terniflora* var. *mandshurica*	Sub flattened	Large, >10 layers	>10 layers	6	4	10	1
*C. urticifolia*	Sub flattened	Large, >10 layers	5-10 layers	5	8	13	4
*C. heracleifolia*	Sub flattened	Large, >10 layers	<5 layers	6	8	14	3
*C. takedana*	Sub flattened	Medium, 5-10 layers	5-10 layers	6	7	13	3
*C. patens*	Flattened	Medium, 5-10 layers	>10 layers	4	2	6	1
*C. brachyura*	V-shaped	Medium, 5-10 layers	<5 layers	5	4	9	2
*C. serratifolia*	Sub flattened/U-shaped	Large, >10 layers	5-10 layers	5	3	8	1
*C. fusca* var. *fusca*	U-shaped	Large, >10 layers	<5 layers	5	2	7	2
*C. fusca* var. *flabellata*	U-shaped	Small, <5 layers	<5 layers	5	5	10	3
*C. fusca* var. *violacea*	Sub flattened	Large, >10 layers	<5 layers	5	2	7	2
*C. calcicola*	Sub flattened/U-shaped	Medium, 5-10 layers	<5 layers	5	4	9	2
*C. koreana*	V-shaped	Small, <5 layers	<5 layers	5	3	8	2
*C. ochotensis*	U-shaped	Medium, 5-10 layers	<5 layers	5	0	5	0

**Notes.**

Abbreviations MVB major vascular bundles IVB interfascicular vascular bundle TVB total vascular bundle VBG vascular bundles in groove

### Petiole outline and upper surface groove

The studied species show considerable variation in their CS petiole outline (Table 2). The species can be divided into three categories based on the shape of their petioles in CS: pentagonal petioles (seven species), U-shaped petioles (nine species), and U-shaped or semi-circular petioles (only *C. terniflora*) (shown in Figs. 3–6). *Clematis hexapetala* and *C. serratifolia* exhibit both pentagonal and semi-circular petioles in CS. Out of the 19 taxa, 13 taxa exhibited two noticeable upper or dorsal surface wings, while six species did not have noticeable wings. Based on the shape of the upper surface groove, the petioles can be categorized as flattened (three species), sub-flattened (11 species), U-shaped (three species), or V-shaped (three species). The formation of the upper surface groove is due primarily to the dorsal surface wings, although some species with inconspicuous wings have a slight groove in the petiole (*C. brevicaudata*, *C. trichotoma*, and *C. heracleifolia*).

### Petiole epidermis and cortex

The epidermis is single-layered and cutinized in all species (Figs. 3–6). The cells are small, rounded, cuboidal, narrow, or slightly elongated. In some places, the continuation of the epidermis is interrupted by the presence of stomata. Stomata are important regulators of gas and water exchange in plants and are usually found in the leaves. They can be found in the stem and petiole but are less prominent in comparison to the leaf. The epidermis is underlain by the cortex, which is 3–5 cells thick. The cortical cells are loosely arranged and parenchymatous with abundant air spaces and are rounded, ovoid, elongated, or irregular in shape. The cortex is collenchymatous above the phloem patches, where the cells are thick-walled and closely packed.

### Vascular bundles

The vascular bundles are of the open, conjoint, collateral type. There is remarkable variation in the number of vascular bundles (ranging from five to 14) among *Clematis* species. The 19 taxa have four to six major vascular bundles and a maximum of eight interfascicular vascular bundles (*C. heracleifolia* and *C. urticifolia*). Twelve species have five major vascular bundles, six species have six, and only *C. patens* has four (Table 2). The major vascular bundles are ovoid with the phloem oriented towards the cortex and the xylem oriented towards the pith. The xylem and phloem are separated by 2-4 layers of cambial cells. Each major vascular bundle is overlain with a cluster of thick-walled fibrous cells, i.e., the phloem fibre cap. The quantity of phloem fibres within the studied species is variable. Based on its height in cell layers, the phloem cap is categorized as large (more than ten cells high), medium (five to ten cells high), or small (less than five cells high). Three species *C. terniflora*, *C. fusca* var. *fusca*, and *C. koreana*, have small fibre caps, while seven and nine species have medium and large fibre caps, respectively. There is a permanent sclerenchymatous strand between the adjacent vascular bundles in all species.

The petiole of each species has a large region of the ground tissue, i.e., the pith. The cells in the pith are thin-walled, rounded, ovoid, or angular and parenchymatous and are comparatively larger than those in the cortex (Figs. 3–6).

### Statistical analysis

**Figure 7:**
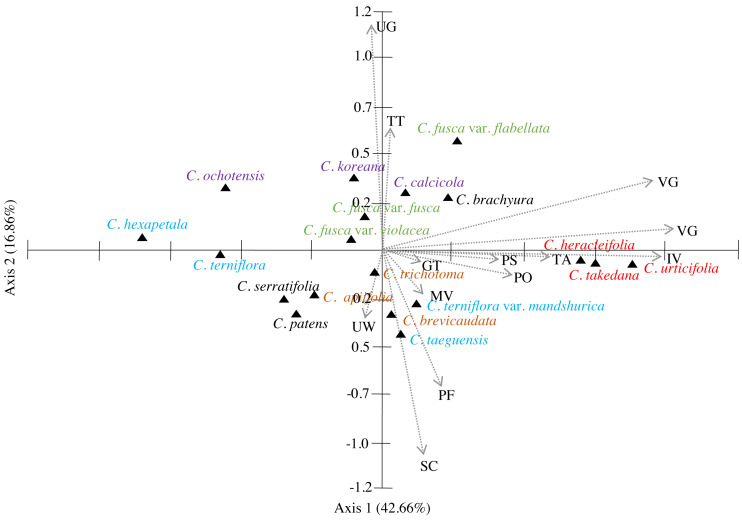
Principal component analysis (PCA) of 13 petiole characters of *Clematis* taxa. PS, petiole surface; TT, trichome type; GT, glandular trichome; TA, trichome abundance; PO, petiole outline in cross-section; UW, upper surface wings; UG, upper surface groove; PF, phloem fiber cap; SC, interfascicular sclerenchyma; MV, major vascular bundles; IV, interfascicular vascular bundle; VB, total vascular bundle; VG, vascular bundles in the groove. Different colour represents different sections of the genus.

**Figure 8:**
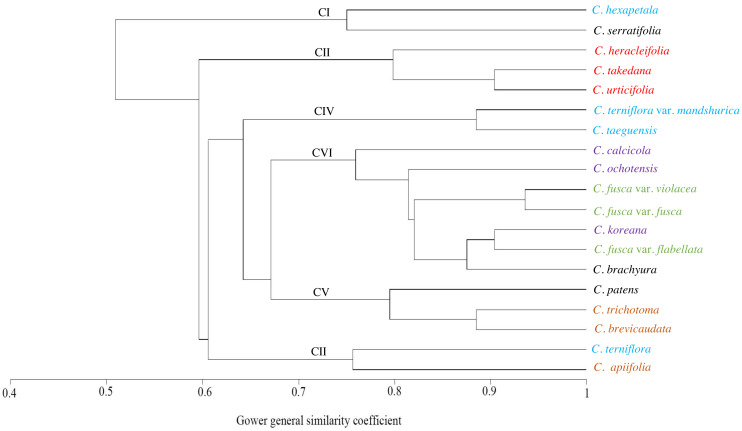
UPGMA cluster analysis based on petiole characters of *Clematis* taxa. Different colours represents different sections of the genus.

The similarities among the species based on the 13 petiole features were revealed using PCA and cluster analysis. The first three components of the PCA explained 74.01% of the total variation in the analysed data. The first axis of the first complete set explained 42.66% of the total variation and showed strong positive loadings for the trichome abundance and the number of vascular bundles (TA, IV, VB, and VG) (Fig. 7). The second axis explained 16.86% of the total variation and showed strong positive loadings for the trichome type and upper surface groove (TT and UG) and strong negative loadings for phloem the fibre cap height and interfascicular sclerenchyma (PF and SC). The cluster analysis based on UPGMA using the Gower similarity coefficient identified six clusters with 18 nodes (Fig. 8). *Clematis fusca* var. *fusca* and *C. fusca* var. *violacea*, representing the first node in the sixth cluster of the phenogram, showed 93.6% similarity in petiole features, whereas *C. hexapetala* and *C. serratifolia*, representing the 18th node in the first cluster in the phenogram, shared only 52.7% similarity in petiole features with the rest of the species.

## Discussion

The genus *Clematis* is morphologically diverse in terms of leaf phyllotaxy, types of compound leaves, and leaflet number. The anatomy of the petiole, therefore, is expected to be equally diverse. Previously, Oh (1971) found variation in the number of vascular bundles in the petiole of some *Clematis* species. Thus, reasonable diversity in petiole anatomical features, including variation in the number of vascular bundles, is certainly useful for the taxonomic treatment of the genus. The overall anatomical organization of the petioles in the investigated species was comparable to that described in Oh (1971). In addition, this study provides a comprehensive anatomical description of the petioles of all Korean *Clematis* species and a discussion of their taxonomic relevance within the genus, which have not been included in previous studies.

The results of this study showed that *Clematis* species can be differentiated based on the petiole indumentum. The presence/absence and/or type of trichomes in the petiole have also been used for species differentiation in other taxa (Solereder, 1908; Metcalfe & Chalk, 1979; Talip et al., 2017). Among the 19 species investigated, *Clematis hexapetala* is the only species with glabrous petioles (although sparsely distributed trichomes can be observed at the base of the leaflets), whereas *C. taeguensis* and *C. serratifolia* have subglabrous or sparsely pubescent indumentum. The remaining species have sparsely or thickly pubescent petioles. In sparsely pubescent taxa such as *C. terniflora*, *C. terniflora* var. *mandshurica*, *C. fusca* var. *fusca*, and *C. fusca* var. *violacea*, the trichomes are restricted to mainly the upper groove region. Additionally, the stem of these taxa is either subglabrous or puberulous only at nodes (Wang & Bartholomew, 2001; Kim, 2017). On the other hand, species such as *C. apiifolia*, *C. heracleifolia*, *C. urticifolia*, and *C. takedana*, which have thickly pubescent petioles, also have heavily pubescent stems and branches (Wang & Bartholomew, 2001; Kadota, 2006; Moon et al., 2013; Kim, 2017). This indicates that the trichomes in the petiole are generally continuous with those on the stem in the *Clematis* species.

Most of the species have both types of trichomes, although glandular trichomes are very scarce and restricted to the upper surface groove of the petiole. We observed both glandular capitate and simple non-glandular trichomes on the leaf surface of most of the investigated *Clematis* species (Ghimire et al. unpublished report). Glandular pubescence on the stem, petiole and leaf of *C. gattingeri* Small has been reported in a few older studies (Svenson, 1941; Dennis, 1978). Petiole features that are considered to be useful for taxonomic discrimination in various taxa (Solereder, 1908; Metcalfe & Chalk, 1979; Dehgan, 1982; Rojo, 1987; Pedri, Hussin & Latiff, 1991; Kamel & Loufty, 2001; Kocsis & Borhidi, 2003; Talip et al., 2016; Talip et al., 2017) are generally neglected in morphological studies of *Clematis* because no recent reports have considered the systematic utility of such pubescence for the infra-generic classifications of this genus (Tamura, 1995; Wang & Li, 2005; Lehtonen, Christenhusz & Falck, 2016; Wang & Bartholomew, 2001; Kadota, 2006; Kim, 2017). In a taxonomic study of *C. gattingeri*, Dennis (1978) confirmed the presence of glandular pubescence on the stem, petiole, and leaf, which had never been observed in other species of subsection *Viornae*. The recognition of *C. gattingeri* as a species has been based primarily on the glandular pubescence and small flower size of Gattinger’s specimens. The results from this study also revealed that the petiole indumentum and types of trichomes appear to have taxonomic value for species delimitation in *Clematis*.

In addition to the surface indumentum, some other petiole features that can contribute to the identification of a particular species in *Clematis* include the petiole outline in CS, the upper surface groove and wings, and the phloem fibre cap. Of these features, the petiole outline and upper surface groove and wings have already been proven to be useful in the taxonomic discrimination of species in some eudicot genera (Kocsis & Borhidi, 2003; Talip et al., 2017; Abeysinghe & Scharaschkin, 2019). Oh (1971) reported pentagonal and/or horseshoe or rounded horseshoe-shaped petioles in nine *Clematis* species, and the results of this study corroborated those reports. We observed that the petiole of *Clematis* in CS is dorsiventral, pentagonal with five visibly and/or weakly represented ridges or semicircular and ridgeless. *Clematis hexapetala* and *C. serratifolia* appear to have both pentagonal and U-shaped petioles, whereas some petioles of *C. terniflora* are semi-circular. Species with pentagonal petioles have conspicuous upper surface wings that form an upper surface groove, while some of the species with U-shaped petioles have inconspicuous upper surface wings. Species with inconspicuous wings such as *C. brevicaudata*, *C. trichotoma*, *C. heracleifolia*, and *C. terniflora*, still form a slight upper surface groove. Of the three taxa *C. fusca* var. *fusca*, *C. fusca* var. *flabellata*, and *C. fusca* var. *violacea*, the first has a pentagonal petiole, while the latter two have U-shaped petioles with noticeable upper wings. Interestingly, these three taxa showed dissimilar upper surface groove characteristics: *C. fusca* var. *violacea* has a sub-flattened groove, whereas *C. fusca* var. *fusca* and *C. fusca* var. *flabellata* have a U-shaped groove. Although reports on such upper wing extensions and adaxial grooves on the petiole are lacking in the literature, our study suggests the possibility of using these features for species identification in *Clematis*.

In petiole anatomy, the vascular system of the petiole has received the most attention (Kocsis & Borhidi, 2003; Talip et al., 2016; Talip et al., 2017 Abeysinghe & Scharaschkin, 2019). According to Hare (1942), various arrangements of vascular bundles in the petiole can be used as diagnostic characteristics in some taxonomic groups. Howard (1962), Howard (1974)) suggested that the vascular structure of the petiole is most useful at the generic level and sometimes at the family level, although the intensity of the taxonomic value may vary from one taxonomic group to another. The *Clematis* petiole showed remarkable consistency in the arrangement of the vascular system, although the studied species differ from each other by the number of major and interfascicular vascular bundles. There are typically five major vascular bundles, which possibly correspond to the five ridges of the petiole; however, *C. patens*, with exclusively U-shaped petioles, has no ridges, wings, or upper surface grooves, and has only four major vascular bundles. In some taxa, such as *C. apiifolia*, *C. taeguensis*, *C. hexapetala*, *C. terniflora* var. *mandshurica*, *C. heracleifolia*, and *C. takedana*, the vascular bundle in the upper groove regions develops in the same way as the vascular bundles in the edges, resulting in a total of six major vascular bundles in these taxa.

Regarding the number of vascular bundles, the results of this study are almost congruent with those of Oh (1971) for *C. apiifolia*, *C. trichotoma*, and *C. koreana* but are slightly different for *C. brachyura* and *C. patens*, in which he described six major and four interfascicular vascular bundles and six major vascular bundles, respectively. Instead, we observed five major and four interfascicular vascular bundles in *C. brachyura* and four major and one or two interfascicular bundles in *C. patens.* We observed variation in the number and position of interfascicular bundles even in the different samples of the same species, including in *C. patens*, thus, this feature may have only slight taxonomic value for discriminating among *Clematis* species. On the other hand, the number and position of the major vascular bundles, which showed remarkable consistency among the investigated samples of all species, may have significant taxonomic value for species discrimination within the genus. At this point, our results suggest that the pre-existing data presented by Oh (1971) on the number of vascular bundles specifically, the number of major bundles in the petioles of *Clematis* species should be corrected.

The cluster analysis based on 13 petiole features generated at least six clusters. Of these, *Clematis serratifolia* and *C. hexapetala* formed the first cluster, which was separate from the rest of the species (Fig. 8). The sixth cluster was the largest comprising seven taxa: *C. calcicola*, *C. ochotensis*, *C. fusca* var. *fusca*, *C. fusca* var. *violacea*, *C. fusca* var. *flabellata*, *C. koreana*, and *C. brachyura*. According to Johnson (1997) and Wang & Li (2005), these seven taxa belong to the sections *Viorna* (*C. fusca* var. *fusca*, *C. fusca* var. *violacea*, *C. fusca* var. *flabellata*), *Atragene* (*C. ochotensis*, *C. calcicola*, and *C. koreana*), and *Pterocarpa* (*C. brachyura*). In a recent phylogenetic classification of the genus, *C. fusca* var. *fusca*, *C. fusca* var. *violacea*, and *C. fusca* var. *flabellata* were classified under clade L; *C. ochotensis*, *C. calcicola*, and *C. koreana* were classified under clade H: and *C. brachyura* was classified under clade K (Lehtonen, Christenhusz & Falck, 2016). Additionally, *C. urticifolia*, *C. takedana*, and *C. heracleifolia*, which made up the third cluster in the UPGMA phenogram in this study, belong to section *Tubulosae* in the Johnson (1997) and Wang & Li (2005) classification and to clade C in Lehtonen, Christenhusz & Falck (2016). According to infrageneric classifications by Tamura (1995), Wang & Li (2005) and Xie, Wen & Li (2011), *C. brachyura* is considered to be closer to section *Flammula* (*C. taeguensis*, *C. hexapetala*, *C. terniflora* var. *mandshurica*, and *C. terniflora*). However, in this study, it remained connected to the *Viorna* and *Atragene* sections. In fact, this uncertainty is a common and typical interpretation for this large genus, as previous morphological and molecular studies also found a similar tendency (Grey-Wilson, 2000; Wang & Li, 2005; Miikeda et al., 2006; Xie, Wen & Li, 2011; Xie & Li, 2012; Lehtonen, Christenhusz & Falck, 2016; Ghimire et al., 2020).

According to PCA, the close affinity of the taxa from section *Tubulosae* is well retained by their proximity in axis 1 (Fig. 7). Previous studies have suggested that neither molecular analyses nor morphological data strongly support the infrageneric classification of *Clematis*. We agree with most authors that performing infrageneric classification of *Clematis* based on morphological characters is extremely difficult. In our analysis, out of the three taxa from section *Viorna*, *C. fusca* var. *flabellata* was grouped with *C. koreana* (section *Atragene*) and *C. brachurya* (section *Pterocarpa*), whereas *C. fusca* var. *fusca* and *C. fusca* var. *violacea* remained together in a separate sub-cluster. The PCA biplot also inferred similar relationships among these three subspecific taxa, with *C. fusca* var. *flabellata* was positioned on the positive side of axis 1, while *C. fusca* var. *fusca* and *C. fusca* var. *violacea* remained closer on the negative side of axis 1. The petioles of *C. fusca* var. *flabellata* differ from those of *C. fusca* var. *fusca* and *C. fusca* var. *violacea* in terms of the trichome abundance, phloem fibre cap height, and number of vascular bundles. In terms of morphology, *C. fusca* var. *flabellata* is an erect herb with ternate leaves, whereas *C. fusca* and *C. fusca* var. *violacea* are woody vines with pinnately foliate leaves. In addition, of the three species in section *Clematis*, *C. trichotoma* and *C. brevicaudata* formed a sub-cluster with *C. patens* (section *Viticella*), while *C. apiifolia* allied with *C. terniflora* (section *Flammula*) in another sub-cluster. The PCA showed a slightly different pattern, as *C. apiifolia* remained closer to *C. serraatifolia* and *C. patens* on the negative side of both axes, whereas *C. brevicaudata* allied with *C. taeguensis* and *C. terniflora* var. *mandshurica* on the positive side of axis 1 and the negative side of axis 2. Morphologically, the petiole of *C. apiifolia* differs from those of the other two species in its trichome abundance, outline in CS, phloem fibre cap, intrafascicular sclerenchyma, and number of vascular bundles. Notably,petiole features alone can be more useful for species delimitation than for distinguishing infrageneric relationships and thus are not indicative of infrageneric classifications. Similar explanations were provided by Ghimire et al. (2020) and Xie & Li (2012) based on the achene morphology and pollen morphology of *Clematis*, respectively.

In conclusion, the presence/absence and abundance of trichomes, petiole outline in CS, upper surface wings and groove, and number of vascular bundles were determined to be useful for species discrimination in Korean *Clematis*. The results of this study also indicated that taxa from the sections *Tubulosae*, *Viorna*, and *Atragene* could be grouped based on the overall similarity of their petiole features. Moreover, we found that many of the petiole characters such as the abundance of trichomes, upper surface wings and grooves, and number of vascular bundles, that were neglected in taxonomic and systematic considerations within and among *Clematis* taxa can be useful for species delimitation. We do not consider these petiole morphological data to be strong enough to provide useful information on infrageneric relationships with the *Clematis* genus; however, our results provide new and interesting insights into these data, which can be used as a source of descriptive and/or diagnostic features for particular taxa in the genus.

##  Supplemental Information

10.7717/peerj.11669/supp-1 Supplemental Information 1 Character and character states of petiole of *Clematis* species

## Additional Information and Declarations

### Data Availability

The following information was supplied regarding data availability: All the raw data used in the statistical analysis are available in the Supplemental File.
